# High reproducibility of lipid core burden measurements with near-infrared spectroscopy and intravascular ultrasound imaging catheter

**DOI:** 10.1016/j.ijcha.2026.101965

**Published:** 2026-06-27

**Authors:** Abdalla Ibrahim, Haroun Butt, Sarosh Khan, Damandeep Kharoud, Qiang Xue, Uzma Sajjad, Guilherme Movio, Gerald Clesham, Ozan M. Demir, Christpher Cook, Evan Ansell, Michael McGarvey, Richard Jones, Grigoris V. Karamasis, John R. Davies, Nilesh Pareek, Thomas R. Keeble

**Affiliations:** a The Essex Cardiothoracic Centre, MSE, Basildon, UK; b Anglia Ruskin School of Medicine & MTRC, Anglia Ruskin University, Chelmsford, UK; c King's College Hospital NHS Foundation Trust, London, UK; d School of Cardiovascular and Metabolic Medicine & Sciences, British Heart Foundation Centre of Excellence, King's College London, UK; e Attikon University Hospital, Athens Medical School, National and Kapodistrian University of Athens, Athens, Greece

**Keywords:** Near-infrared spectroscopy, Intravascular ultrasound, Lipid core burden index, maxLCBI4mm, Plaque vulnerability, Reproducibility

## Abstract

**Background:**

Near-infrared spectroscopy combined with intravascular ultrasound (NIRS–IVUS) allows simultaneous assessment of plaque composition and vessel structure. The lipid core burden index (LCBI) and the maximum lipid core burden index over 4 mm (_max_LCBI_4mm_) are established markers of lipid-rich and potentially vulnerable plaques. However real-world reproducibility data for these indices using the contemporary Dualpro™ NIRS–IVUS catheter is unknown. This study aimed to evaluate the reproducibility of LCBI and _max_LCBI_4mm_ across repeated NIRS–IVUS pullbacks performed within the same coronary segment using the current-generation catheter system.

**Methods:**

This single-center study included consecutive patients who underwent percutaneous coronary intervention (PCI) with adjunctive NIRS–IVUS imaging and had repeated pullbacks of the same coronary segment during the procedure. LCBI and _max_LCBI_4mm_ were recorded for each pullback. Reproducibility was assessed using Spearman correlation and intraclass correlation coefficients (ICC), and consistency for the clinically relevant thresholds of _max_LCBI_4mm_ was evaluated.

**Results:**

A total of 87 paired pullbacks were analyzed (37 pre-PCI, 15 post-lesion preparation, and 35 post-stenting). LCBI demonstrated excellent reproducibility (ρ = 0.95; ICC = 0.95) and _max_LCBI_4mm_ also showed strong reproducibility (ρ = 0.90; ICC = 0.91). The threshold of _max_LCBI_4mm_ > 400 was concordant between runs in 90% of cases, and the post-stent threshold of >200 showed concordance in 89% of cases.

**Conclusion:**

The contemporary Dualpro™ NIRS–IVUS catheter provides highly reproducible measurements of both LCBI and _max_LCBI_4mm_. These findings support the reliability of NIRS–IVUS for identifying lipid-rich plaques and strengthen its suitability for research and clinical applications focused on plaque vulnerability.

## Highlights

• Contemporary NIRS–IVUS provides highly reproducible lipid plaque quantification.
• LCBI and maxLCBI4mm remain consistent across repeated coronary pullbacks.
• Reproducibility is preserved before PCI and after stent implantation.
• Clinically relevant lipid thresholds show high concordance between runs.
• Supports NIRS–IVUS for serial imaging and vulnerable plaque studies.

## 1 Introduction

In contemporary percutaneous coronary intervention (PCI), the role of intracoronary imaging has become increasingly central to both procedural planning and optimization [1], [2]. Technologies such as intravascular ultrasound (IVUS), optical coherence tomography (OCT), and near-infrared spectroscopy (NIRS) provide essential insights into plaque morphology, vessel sizing, and stent optimization, which are often not adequately visualized on angiography alone [1], [2]. Among these, hybrid NIRS-IVUS imaging offers a unique advantage by simultaneously characterizing plaque composition and coronary anatomy, combining NIRS for spectroscopic analysis of plaque lipid burden with IVUS for structural assessment of plaque morphology and vessel architecture [3]. The Makoto™ Intravascular Imaging System and its accompanying Dualpro™ NIRS-IVUS catheter (Nipro, Japan) represents the only IVUS-based intracoronary imaging modality capable of simultaneously detecting and quantifying lipid-rich plaques while measuring plaque burden and lumen area in the coronary arteries [4]. The lipid core burden index (LCBI) is a quantitative measure of the lipid content within a coronary segment, calculated as the fraction of yellow pixels, indicative of lipid, in the NIRS chemogram multiplied by 1000. The maximum lipid core burden index over 4 mm (_max_LCBI_4mm_) refers to the highest LCBI value found within any contiguous 4-mm segment of the imaged artery [4]. A _max_LCBI_4mm_ > 400 has been shown to be associated with a significantly increased risk of future major adverse cardiac events (MACE), independent of traditional risk factors and plaque burden [5], [6]. More recently, post-stent studies using NIRS-IVUS have demonstrated that a residual _max_LCBI_4mm_ > 200 within the stented segment is associated with a higher risk of target lesion failure during long-term follow-up [7]. This finding suggests that retained lipid-rich plaque behind or adjacent to stents may contribute to recurrent events. These thresholds have therefore become clinically relevant reference points for identifying high-risk plaques before and after PCI, underscoring the importance of ensuring the reproducibility of these measurements with the contemporary NIRS–IVUS system. Previous studies assessing the reproducibility of LCBI as measured by NIRS demonstrated high reproducibility in duplicate pullbacks performed in the same coronary segments, showing excellent correlation and strong agreement between repeated measurements [8], [9]. However, these studies were conducted using earlier generations of NIRS catheters without combined IVUS imaging, and prior to the introduction of the _max_LCBI_4mm_ metric in 2013 and an understanding of its clinical significance [10], [11]. With the increasing interest and emerging data on the importance of identifying vulnerable plaques [12], and the availability of the contemporary Dualpro™ NIRS-IVUS catheter, there is a need to reassess the reproducibility of both LCBI and _max_LCBI_4mm_ using this new system. The primary aim of this study was to evaluate the reproducibility of LCBI and _max_LCBI_4mm_ across repeated imaging runs within the same coronary arteries using the contemporary NIRS-IVUS catheter in a real-life clinical setting. Specifically, the study aimed to quantify inter-run variability of these indices, assess reproducibility at different procedural stages (pre-PCI and post-stent deployment), and determine whether values exceeding clinically relevant thresholds (e.g., _max_LCBI_4mm_ > 400 and > 200) remain consistent across repeated measurements.

## 2 Methods

### 2.1 Study design and population

This was a single-centre, observational study analyzing routinely acquired intracoronary NIRS–IVUS data between November 2023 and September 2024. Consecutive patients with acute or chronic coronary syndromes undergoing PCI with clinically indicated repeated NIRS-IVUS imaging were included.

### 2.2 Procedural technique and imaging protocol

**Fig. 1:**
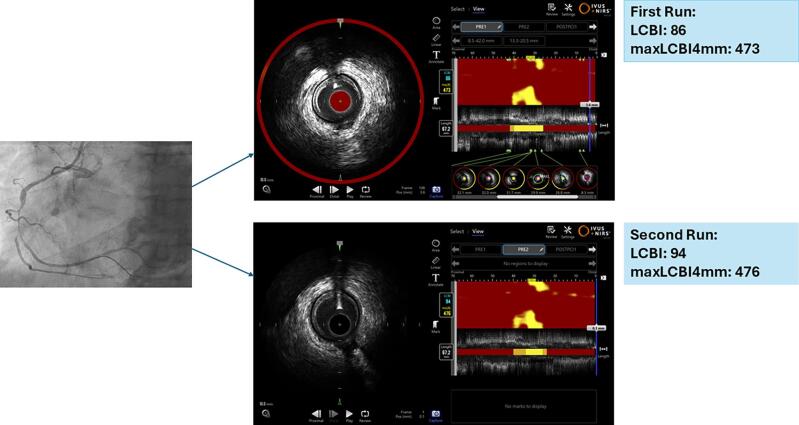
Example of a NIRS-IVUS repeated pullbacks in a single coronary segment. LCBI: lipid core burden index, maxLCBI4mm: maximum lipid core burden index over 4 mm.

All procedures were performed via the radial artery using a standard 6F guiding catheter and all patients received appropriate anticoagulation with heparin. Imaging was performed using the Makoto™ Intravascular Imaging System and the accompanying Dualpro™ NIRS-IVUS catheter (Nipro, Japan). The catheter was advanced over a standard 0.014 coronary guidewire to a stable distal reference segment beyond the lesion of interest, at least 5 mm distal to the target lesion and in a segment with plaque burden <50%, where feasible. In STEMI cases, NIRS–IVUS imaging was performed after restoration of sufficient antegrade flow to allow safe catheter passage. Where clinically required, this included thrombus aspiration and balloon predilatation before imaging, at the operator's discretion. Automated NIRS-IVUS pullbacks were performed at a constant speed of 2 mm/s using the system's motorized pullback feature. Immediately after the completion of the first pullback, the Dualpro™ catheter was automatically repositioned by the system to the same distal starting point without moving the imaging setup, ensuring that the second pullback was performed over the identical coronary segment. This process was repeated following stent deployment, as clinically indicated. Each paired pullback runs were performed by the same operator and for each pullback run, the Makoto™ Intravascular Imaging System automatically calculated LCBI and the _max_LCBI_4mm_. **(**Fig. 1**).** The _max_LCBI_4mm_ value was recorded as the highest LCBI within any contiguous 4-mm segment of the analyzed pullback, as automatically identified by the Makoto™ system, and was not required to correspond to the minimum lumen area or maximum plaque burden site. Data was reviewed at the time of the PCI procedure, with no additional post-procedure data collection undertaken.

### 2.3 Statistical analysis

Data distribution was assessed using the Shapiro–Wilk test and non-parametric methods were applied where appropriate. Continuous variables were expressed as median (interquartile range) (IQR). Reproducibility between repeated runs was assessed using Bland–Altman analysis to determine mean bias and limits of agreement, intraclass correlation coefficients (ICC) to assess absolute agreement, and Spearman's rank correlation coefficient (ρ) to evaluate the strength of the association between measurements. The consistency of detecting the clinically relevant cutoff of _max_LCBI_4mm_ > 400 (in the pre-PCI stage) and _max_LCBI_4mm_ > 200 (in the post-stent stage) between runs was also examined. Differences in reproducibility between plaques stratified by lipid burden—specifically those with _max_LCBI_4mm_ above or below the pre-PCI threshold of 400 and the post-stent threshold of 200—were assessed using Fisher's z-test to compare correlation coefficients, while overlap of the 95% confidence intervals for ICC values was used to evaluate agreement between groups. Statistical analyses were performed using Jamovi (version 2.6.13, The Jamovi Project, Sydney, Australia).

### 2.4 Sample size considerations

Sample size adequacy was assessed using standard methods for estimating reliability coefficients. Assuming an expected ICC of 0.90 based on previous NIRS reproducibility studies [8], [9], a minimum of 80 paired measurements was required to detect an ICC significantly greater than 0.75 with 90% power and a two-sided α of 0.05. The present study included 87 paired NIRS–IVUS runs, exceeding this requirement and providing sufficient sample size for reproducibility analyses.

### 2.5 Ethics and funding considerations

This study was conducted under approval from a UK Health Research Authority (Reference 26/HRA/0577), which permits the retrospective analysis of anonymized routinely collected clinical data without the requirement for individual patient consent. The study was performed in accordance with the principles of the Declaration of Helsinki. This study received no specific funding, and no external funding body had any role in the study design; data collection, analysis, or interpretation; the decision to publish; or the preparation of the manuscript.

## 3 Results

### 3.1 Study population and imaging runs

Data from 43 patients (48 vessels) were collected and analyzed. The baseline, clinical, angiographic, and procedural characteristics are summarized in Table 1 and Table 2. A total of 87 paired NIRS-IVUS runs were analyzed, comprising 37 (43%) pre-PCI runs, 15 (17%) post-lesion preparation runs, and 35 (40%) post-stenting runs.

**Table 1:** Clinical characteristics of the study population (*n* = 43).

Variable	Value
Age (years)^a^	72 ± 11.0
Men, n (%)	35 (82)
*Ethnicity, n (%)*	
White	38 (89)
Body mass index (kg/m^2^)^a^	26.5 ± 1.8
*Past medical history, n (%)*	
Previous myocardial infarction	17 (39)
Previous PCI	12 (27)
Hypertension	28 (65)
Hypercholesterolaemia	27 (62)
Diabetes mellitus	12 (27)
Family history of IHD	7 (16)
Current or ex-smoker	28 (65)
Prior stroke/TIA	1 (2)
CKD (GFR < 60)	6 (14)
Peripheral vascular disease	3 (7)
*Medication, n (%)*	
Statin therapy	18 (42)
*Clinical presentation, n (%)*	
STEMI	19 (44)
Non-ST-segment elevation ACS	11 (25)
Chronic coronary syndrome	13 (31)
*Lipid profile (mmol/L)*^*a*^	
Total cholesterol	4.8 ± 1.2
LDL cholesterol	2.7 ± 1.1

ᵃ Mean ± standard deviation.

ACS = acute coronary syndrome; CKD = chronic kidney disease; GFR = glomerular filtration rate; IHD = ischaemic heart disease; LDL = low-density lipoprotein; PCI = percutaneous coronary intervention; STEMI = ST-segment elevation myocardial infarction; TIA = transient ischaemic attack.

**Table 2:** Procedural and angiographic characteristics.

Variable	Value
*Target vessel, n (%)*	
LAD	30 (62)
Cx	8 (17.5)
RCA	8 (17.5)
*Angiographic characteristics*^*a*^	
Artery length scanned (mm)	62.1 ± 23.6
Plaque burden (%)	67.7 ± 15.8
Lesion length (mm)	32.2 ± 19.3
Minimal lumen area (mm^2^)	3.1 ± 1.1
*Stent characteristics*^*a*^	
Number of stents	1.19 ± 0.52
Total stent length (mm)	32.6 ± 19.8
Largest nominal stent diameter (mm)	3.32 ± 0.78
*Lesion preparation, n (%)*	
Non-compliant balloon	22 (52.4)
Semi-compliant balloon	13 (31.0)
Intravascular lithotripsy	3 (7.1)
Atherectomy (rotational)	5 (11.9)

ᵃ Mean ± standard deviation.

Cx = circumflex artery; LAD = left anterior descending artery; RCA = right coronary artery.

### 3.2 Overall reproducibility of LCBI and _max_LCBI_4mm_

**Fig. 2:**
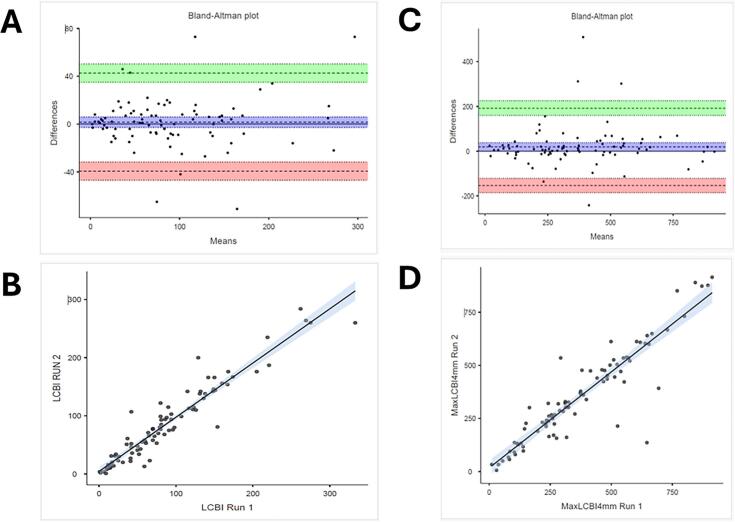
Bland-Altman and Scatter Plots Demonstrating Overall Reproducibility of LCBI (A&B) and maxLCBI4mm (C&D). LCBI: lipid core burden index, maxLCBI4mm: maximum lipid core burden index over 4 mm.

Across all paired imaging runs (*n* = 87), the median LCBI was 77 (IQR 86) for the first run and 76 (IQR 92) on the second, showing an excellent consistency and agreement between repeated measurements (Spearman's ρ = 0.948, *p* < 0.001; ICC = 0.95, 95% CI 0.93–0.97, p < 0.001). The median _max_LCBI_4mm_ was 325 (IQR 291) for the first run and 321 (IQR 283) on the second, showing excellent consistency and agreement (Spearman's ρ = 0.90, p < 0.001; ICC = 0.91, 95% CI 0.87–0.94, *p* < 0.001). **(**Fig. 2**).**

### 3.3 Reproducibility in Pre-PCI stage

**Fig. 3:**
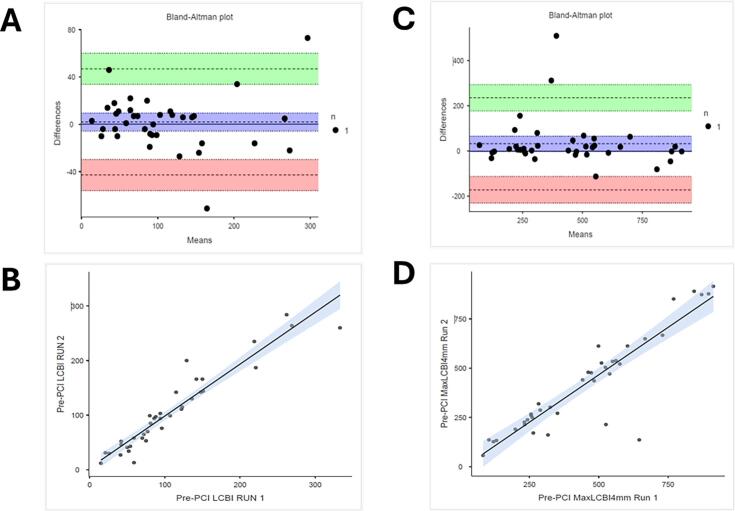
Bland-Altman and Scatter Plots Demonstrating Reproducibility of LCBI (A,B) and maxLCBI4mm (C,D) in the Pre-PCI Stage. LCBI: lipid core burden index, maxLCBI4mm: maximum lipid core burden index over 4 mm, PCI: percutaneous coronary intervention.

When analyzed by procedural stage, reproducibility remained high. In the pre-PCI group (*n* = 37), the median LCBI was 88 (IQR 77) for the first run and 94 (IQR 90) for the second, showing excellent consistency and agreement (Spearman's ρ = 0.96, *p* < 0.001; ICC = 0.95, 95% CI 0.91–0.97, p < 0.001). The corresponding median _max_LCBI_4mm_ was 468 (IQR 316) for the first run and 378 (IQR 320) for the second, demonstrating good consistency and agreement (Spearman's ρ = 0.85, *p* < 0.001; ICC = 0.88, 95% CI 0.80–0.93, p < 0.001). **(**Fig. 3**).**

### 3.4 Reproducibility in post-stent stage

**Fig. 4:**
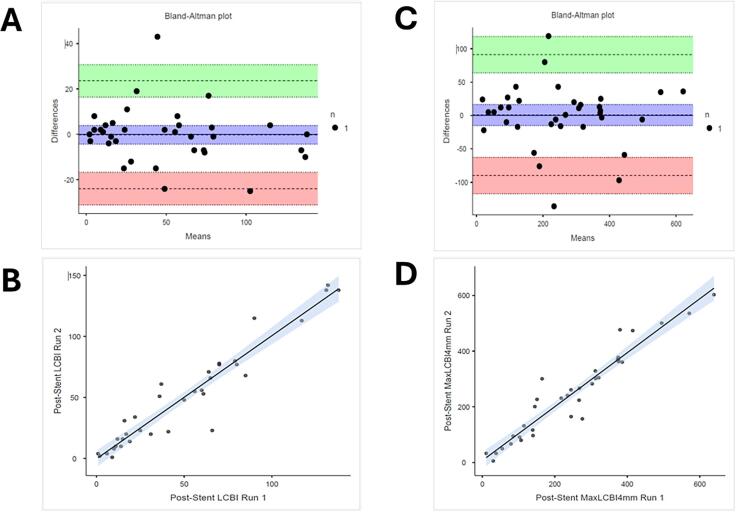
Bland-Altman and Scatter Plots Demonstrating Reproducibility of LCBI (A,B) and maxLCBI4mm (C,D) in the Post-Stenting Stage. LCBI: lipid core burden index, maxLCBI4mm: maximum lipid core burden index over 4 mm.

Similarly, in the post-stenting group (*n* = 35), the median LCBI was 41 (IQR 54.5) for the first run and 48 (IQR 58) for the second, again showing excellent consistency and agreement (Spearman's ρ = 0.95, *p* < 0.001; ICC = 0.94, 95% CI 0.89–0.97, p < 0.001). The median _max_LCBI_4mm_ was 245 (IQR 247) for the first run and 241 (IQR 255) on the second, showing excellent consistency and agreement (Spearman's ρ = 0.96, p < 0.001; ICC = 0.93, 95% CI 0.88–0.96, *p* < 0.001). **(**Fig. 4**).**

### 3.5 Reproducibility of the clinically relevant pre-PCI _max_LCBI_4mm_ > 400 threshold

**Fig. 5:**
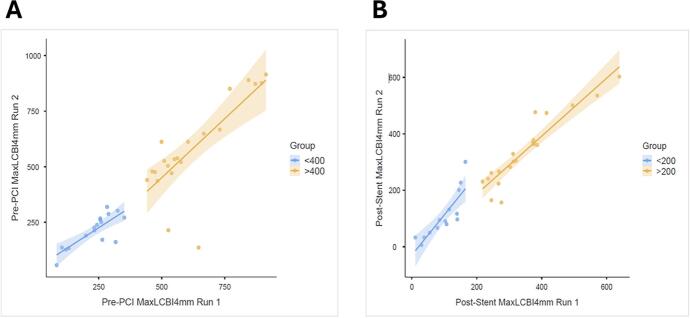
Scatter plots demonstrating the reproducibility of clinically relevant maxLCBI4mm thresholds: (A) pre-PCI cutoff of 400 and (B) post-stent cutoff of 200. LCBI: lipid core burden index, maxLCBI4mm: maximum lipid core burden index over 4 mm, PCI: percutaneous coronary intervention.

When the pre-PCI runs were stratified by lipid burden, reproducibility was similar between plaques with _max_LCBI_4mm_ > 400 and those <400. For lesions with _max_LCBI_4mm_ > 400 (*n* = 21), the median _max_LCBI_4mm_ was 562 (IQR 220) for the first run and 534 (IQR 191) for the second, showing good consistency and agreement (Spearman's ρ = 0.76, *p* < 0.001; ICC = 0.72, 95% CI 0.41–0.88, p < 0.001). For plaques with _max_LCBI_4mm_ < 400 (*n* = 17), the median values were 256 (IQR 84) and 226 (IQR 106) for runs 1 and 2, respectively, also showing good consistency and agreement (Spearman's ρ = 0.75, p < 0.001; ICC = 0.77, 95% CI 0.45–0.91, p < 0.001). A Fisher's z-test comparing the correlation coefficients between groups showed no significant difference (z = 0.04, *p* = 0.97), and the overlapping ICC confidence intervals further confirmed that reproducibility was comparable between high- and low-lipid burden segments. The clinically relevant threshold of _max_LCBI_4mm_ > 400 was concordant between runs in 90% of cases, with 19 of 21 lesions exceeding this threshold on both pullbacks, while 100% of plaques with _max_LCBI_4mm_ < 400 on the first run remained below this cutoff on the second. **(**Fig. 5A**).**

### 3.6 Reproducibility of the clinically relevant post-stent _max_LCBI_4mm_ > 200 threshold

Following stent implantation, reproducibility of _max_LCBI_4mm_ measurements remained high across repeated pullbacks. When segments were stratified by lipid burden using the post-PCI cutoff of 200, both high- and low-lipid plaques showed comparable measurement stability. In lesions with _max_LCBI_4mm_ > 200 (*n* = 22), the median _max_LCBI_4mm_ was 303 (IQR 118) for the first run and 302 (IQR 114) for the second, demonstrating strong consistency and agreement (Spearman's ρ = 0.87, *p* < 0.001; ICC = 0.88, 95% CI 0.73–0.95, p < 0.001). For plaques with _max_LCBI_4mm_ < 200 (*n* = 13), the median values were 115 (IQR 67) and 97 (IQR 63) for runs 1 and 2, respectively, also showing good reproducibility (Spearman's ρ = 0.83, p < 0.001; ICC = 0.85, 95% CI 0.62–0.94, p < 0.001). A Fisher's z-test comparing correlation coefficients between groups showed no significant difference (z = 0.26, *p* = 0.79), confirming similar reproducibility across the lipid spectrum. Classification above or below the 200 threshold was concordant in 88.6% of cases with 31 of 35 lesions remaining within the same category on repeat imaging. **(**Fig. 5B**).**

## 4 Discussion

This study demonstrates that NIRS-IVUS imaging using the contemporary Dualpro™ catheter provides highly reproducible measurements of both LCBI and _max_LCBI_4mm_. Across repeated pullbacks in the same coronary segments, strong correlations and excellent intraclass agreement were observed for both indices, with consistent findings before and after stent implantation.

The application of intra-coronary imaging for guidance of percutaneous coronary intervention for complex lesion subsets has Level 1 A recommendation in both chronic and acute coronary syndromes [13], [14]. While IVUS and OCT are now in routine clinical use, they lack the ability to reproducibly quantify coronary lipid burden, where the _max_LCBI_4mm_ metric has been associated with subsequent MACE and is increasingly used to identify vulnerable plaques in both observational studies and interventional trials [[6], [15]]. NIRS-IVUS is hence expected to play an increasing role in PCI guidance, evaluation of lipid-rich plaque and the effects of targeted systemic preventive therapies, such as lipid-lowering agents, or interventional local strategies including stenting and drug coated balloons (DCBs). Recent evidence has renewed interest in whether treating non-obstructive high-risk plaques, identified by NIRS-IVUS imaging in particular, may reduce future events. The PREVENT trial showed that preventive stenting of lipid-rich, non–flow-limiting plaques reduced MACE, suggesting that plaque stabilization with a stent may benefit selected patients [16]. DCBs on the other hand, remain an established strategy for in-stent restenosis and de novo lesions in specific settings, but evidence supporting their use for vulnerable plaques remains limited [17]. Other local approaches, such as cryotherapy, remain investigational, with only preclinical evidence suggesting possible plaque-stabilizing effects [18], [19]. This highlights the ongoing importance of accurate, valid and reproducible identification of lipid-rich plaques in contemporary practice.

Earlier reproducibility studies conducted by Garcia et al. and Abdel-Karim et al. evaluated duplicate NIRS pullbacks using the first-generation, spectroscopy-only catheter. Those studies also showed excellent correlation for LCBI between runs, establishing the robustness of the NIRS signal [8], [9]. However, these studies preceded the introduction of the _max_LCBI_4mm_ parameter and were performed before integration of NIRS with IVUS in a single catheter [20]. The present study extends those findings to the contemporary Dualpro™ NIRS-IVUS system and provides the first validation of _max_LCBI_4mm_ reproducibility, confirming that this metric, which is now widely adopted as a marker of plaque vulnerability, is measured consistently with the contemporary imaging system. Importantly, the clinically relevant threshold of _max_LCBI_4mm_ > 400 in the pre-PCI setting was concordant in 90% of cases, and no segment below this cutoff crossed above it on repeat imaging. Similarly, the post-stent threshold of _max_LCBI_4mm_ > 200 was highly stable, with nearly all lesions remaining above or below the cutoff on both runs. Together, these findings confirm that lipid quantification with the current generation of NIRS-IVUS provides reproducible lipid quantification across repeated pullbacks and ensures that serial changes observed in serial imaging or treatment studies reliably reflect biological variation rather than measurement variability. In this context, our findings support the use of NIRS-IVUS as a reliable platform for both research and therapy focused studies.

The study population represents a standard real-world PCI cohort, which supports the relevance of these reproducibility findings to everyday clinical practice. The majority were older male patients with high prevalence of common cardiovascular risk factors such as hypertension, hypercholesterolemia, and smoking history. A notable number had previous myocardial infarction or prior PCI, indicating established coronary artery disease (CAD). The clinical presentations included both acute and chronic coronary syndromes, also reflecting routine indications for PCI. Approximately half of the patients were receiving statin therapy, and the lipid profile was typical for patients with established CAD receiving ongoing secondary prevention therapy and hence the findings are relevant to routine clinical practice [21].

The findings of this study have important implications for future studies and potentially clinical practice. Clinically, the reproducibility of these measurements supports the use of NIRS–IVUS for identifying lipid-rich plaques and for future studies assessing plaque modification in response to intensive lipid-lowering or other plaque-stabilizing therapies; however, outcome-based studies are required before these measurements can be used to guide treatment escalation in routine practice. Systemic therapies continue to be the mainstay of plaque modification. Intensive lipid-lowering agents, including statins, PCSK9 inhibitors, and inclisiran, have previously been shown to stabilize plaque structure and reduce lipid burden [18]. Ongoing trials, such as VICTORION-PLAQUE, will help clarify their effect on plaque composition in patients with non-obstructive coronary disease according to non-invasive imaging [22]. We propose that establishing the reproducibility of NIRS-derived indices with the current catheter generation provides an important methodological foundation for the invasive assessment of lipid-rich plaque. Reliable lipid quantification will now enable interpretation of serial changes in plaque composition with greater confidence and may support future studies evaluating plaque modification with intensive lipid-lowering or other targeted therapies.

### 4.1 Limitations

This study has several limitations including that it was conducted at a single center and included a modest sample size, which may limit the generalizability of the findings. In addition, the repeated pullbacks were not analyzed by an independent core laboratory, and formal inter- and intra-observer variability was not assessed. Although LCBI and _max_LCBI_4mm_ were automatically generated by the Makoto™ system, independent core-laboratory analysis would provide further validation, particularly in multicenter studies. All repeated pullbacks were performed during the same PCI procedure, and therefore short-term biological variability could not be assessed. In STEMI cases, any lesion manipulation required to restore flow or permit safe catheter passage may have altered the baseline lipid map, and this should be considered when interpreting pre-PCI measurements. Finally, while the study focused on reproducibility, it did not evaluate the diagnostic or prognostic performance of the NIRS-IVUS indices in this cohort, and it did not include clinical outcome data. Therefore, the findings support the reliability of NIRS–IVUS lipid burden measurement but should not be interpreted as evidence that NIRS–IVUS-guided treatment improves clinical outcomes.

## 5 Conclusion

Our study confirms that the contemporary Dualpro™ NIRS-IVUS catheter provides highly reproducible measurements of both LCBI and _max_LCBI_4mm_ across repeated pullbacks within the same coronary segments. The consistency of lipid quantification using the current NIRS-IVUS system supports its use as a reliable imaging tool for identifying and monitoring lipid-rich plaques and provides a robust methodological foundation for future studies investigating plaque vulnerability and treatment effects.

## CRediT authorship contribution statement

**Abdalla Ibrahim:** Writing – review & editing, Writing – original draft, Formal analysis, Data curation, Conceptualization. **Haroun Butt:** Writing – review & editing, Writing – original draft, Data curation. **Sarosh Khan:** Writing – review & editing, Writing – original draft. **Damandeep Kharoud:** Writing – review & editing, Data curation. **Qiang Xue:** Writing – review & editing, Data curation. **Uzma Sajjad:** Writing – review & editing, Data curation. **Guilherme Movio:** Writing – review & editing, Data curation. **Gerald Clesham:** Writing – review & editing. **Ozan M. Demir:** Writing – review & editing, Writing – original draft. **Christpher Cook:** Writing – review & editing, Writing – original draft. **Evan Ansell:** Writing – review & editing, Writing – original draft. **Michael McGarvey:** Writing – review & editing. **Richard Jones:** Writing – review & editing, Methodology. **Grigoris V. Karamasis:** Writing – review & editing. **John R. Davies:** Writing – review & editing. **Nilesh Pareek:** Writing – review & editing, Methodology, Conceptualization. **Thomas R. Keeble:** Writing – review & editing, Methodology, Data curation, Conceptualization.

## Declaration of competing interest

The authors declare that they have no known competing financial interests or personal relationships that could have appeared to influence the work reported in this paper.
